# Anticancer Inhibitors

**DOI:** 10.3390/molecules27144650

**Published:** 2022-07-21

**Authors:** Alessandra Ammazzalorso, Marialuigia Fantacuzzi

**Affiliations:** Department of Pharmacy, “G. d’Annunzio” University of Chieti-Pescara, Via dei Vestini 31, 66100 Chieti, Italy

## 1. Introduction

Cancer is a multifactorial disorder caused by several aberrations in gene expression that generate a homeostatic imbalance between cell division and death. Because of the worldwide increasing burden and the complexity of the mechanisms involved, considerable efforts have been devoted to cancer management. Many chemotherapeutics have been developed, but most of them have failed in cancer treatment. Antitumor drugs can be divided in non-specific (cytotoxic) drugs and specific drugs (targeted). Due to the inability of non-specific drugs to selectively target tumor cells, targeted therapy has grown more in recent years, allowing researchers to identify drugs characterized by a high specificity towards receptors and enzymes involved in cancer proliferation [1,2,3,4,5,6,7]. Since multiple pathogenetic mechanisms are involved in the development of cancer, the characterization of different types of cancers, which distinguishes them from healthy cells and other cancers, allows for the identification of specific targets for each individual tumor.

The Special Issue “Anticancer Inhibitors” covers twelve contributes (nine original research papers and three reviews). As guest editors, we briefly report an overview of these contributions.

## 2. Results

The roles of cyclin-dependent kinases (CDKs) in different cancers allows for targeting specific kinases to obtain a selective action in the cell cycle and gene transcription. In the last years, CDK4/6 inhibitors revealed a therapeutic role for the treatment of breast cancer. Structural modifications of the three FDA-approved CDK4/6 inhibitors furnished novel molecules currently under clinical investigation as antitumor drugs. The novel generation of PROTACs (proteolysis targeting chimeras) has also been reviewed, allowing for a selective degradation of CDK4 or CDK6 by varying the chemical structures of inhibitors and linkers [8].

Al-Salem et al. synthesized a series of isatin-hydrazones with cytotoxic effects against MCF-7 and A2780 cell lines. Structure–activity relationship studies highlighted the structural modifications mainly responsible for the CDK2 IC_50s_ nanomolar range activity. In silico ADME demonstrated the recommended drug likeness properties, while computational predictions of the binding mode confirmed type II ATP competitive inhibition [9].

Phosphatidylinositide-3-kinase (PI3K)/Akt signaling pathway inhibitors have undergone pre-clinical evaluation as a promising therapy for cancer treatment; the combination use of LY294002 and tamoxifen in breast cancer MCF-7 cells was indagated by Abdallah and coworkers. A synergistic cytotoxic effect of the combination, achieved by the induction of apoptosis and cell cycle arrest through cyclin D1, pAKT, caspases, and Bcl-2 signaling pathways, was found helpful to develop novel and effective therapeutic combination against breast cancer and reduce the toxicity and resistance of LY294002 and tamoxifen [10].

Konkol’ová et al. synthesized novel tacrine–coumarin hybrids as inhibitors of topoisomerase, and enzyme involved in DNA metabolism. Novel compounds inhibit the metabolic activity of A549 cell line in a time- and dose-dependent manner, increase the accumulation of cells in the G0/G1 phase, and topoisomerase I inhibition was confirmed as the mechanism of action of this class of hybrids [11].

Tanuma et al. synthesized novel azaindole–piperidine or azaindole–piperazine to develop effective and safer (no gastrointestinal symptoms and thrombocytopenia) anticancer nicotinamide phosphoribosyltransferase (NAMPT) inhibitors [12].

Tilayov et al. combined rational and combinatorial engineering approaches for transforming dimeric stem cell factor (SCF) into ligands with different agonistic potencies by engineering variants with a reduced dimerization potential and an increased affinity for c-Kit. The combinatorial site-directed engineering of both ligand–ligand and ligand–receptor interactions provides the means to generate improved therapeutic mediators and to gain insights into the dynamics of receptor tyrosine kinases (RTK)–ligand interactions [13].

The involvement of Cathepsin K in non-small-cell lung cancer has been investigated by Yang et al. through in vitro experiments of cell proliferation, migration, and invasion in human cell line A549. The results showed that Cathepsin K was overexpressed, promoting the proliferation, migration, invasion, and activation of the mammalian target of the rapamycin (mTOR) signaling pathway [14]. 

The review by Chen collects the literature evidence about both pro-oncogenic and tumor-suppressive effects of ZMYND8 (zinc finger myeloid, nervy, and deformed epidermal autoregulatory factor 1-type containing 8) in various types of cancer [15]. 

Rimpelová et al. studied the effect of statins on the expression of genes, whose products are implicated in cancer inhibition. The study on MiaPaCa-2 pancreatic cancer cells analyzes the genes involved in the metabolism of lipids and steroids that were affected by statin treatment [16]. 

The pivotal role played by natural products in the discovery and development of novel anticancer agents is well known [17]. The anticancer effect of saponins from tea (*Camellia sinensis*) was investigated by Wang et al. The extracted saponins decreased cell viability and induced morphological changes in OVCAR-3 cells. The autophagic effect occurred independently from Akt/mTOR/p70S6K pathway signaling, but it is linked to ERK activation and ROS generation [18]. A further evaluation on a high purity standardized saponin extract, namely, Baiye No.1 tea flower saponin, demonstrated its potential to be used as a nutraceutical for the prevention and treatment of ovarian cancer [19]. 

Franzyk and Christensen reviewed the recent literature reports on advanced prodrug concepts targeting toxins to cancer tissues. These strategies include antibody-directed enzyme prodrug therapy (ADEPT), gene-directed enzyme prodrug therapy (GDEPT), lectin-directed enzyme-activated prodrug therapy (LEAPT), and antibody-drug conjugated therapy (ADC). In addition, recent examples of protease-targeting chimeras (PROTACs) were also analyzed and discussed; these methods involve ubiquitination enzyme complexes that undergo proteolytic degradation to release the drug. Overall, these innovative strategies of tumor targeting may lead to future new anticancer drugs that are urgently needed for. However, many of these recently developed targeting principles remain to result in approved drugs, which emphasizes the need for further research [20]. 

## 3. Conclusions

This collection contributes to improving the knowledge on anticancer inhibitors, focusing on the synthesis and evaluation of novel compounds able to inhibit enzymes involved in tumorigenesis and proliferation, the role of transcription factors, the use of natural molecules as lead compounds for anti-cancer drug development, and, finally, the search for innovative strategies to overcome pharmacokinetic limitations.
